# Long‐term follow‐up of a racially and ethnically diverse population of men with localized prostate cancer who did not undergo initial active treatment

**DOI:** 10.1002/cam4.3471

**Published:** 2020-09-23

**Authors:** Jeff M. Slezak, Stephen K. Van Den Eeden, Kimberly L. Cannavale, Gary W. Chien, Steven J. Jacobsen, Chun R. Chao

**Affiliations:** ^1^ Department of Research and Evaluation Kaiser Permanente Southern California Pasadena CA USA; ^2^ Division of Research Kaiser Permanente Northern California Oakland CA USA; ^3^ Department of Urology Los Angeles Medical Center Kaiser Permanente Southern California Los Angeles CA USA

**Keywords:** long‐term outcomes, metastasis, prostate cancer, prostate cancer‐specific mortality, race and ethnicity, survival, untreated prostate cancer

## Abstract

**Background:**

There is limited research on the racial/ethnic differences in long‐term outcomes for men with untreated, localized prostate cancer.

**Methods:**

Men diagnosed with localized, Gleason ≤7 prostate cancer who were not treated within 1 year of diagnosis from 1997–2007 were identified. Cumulative incidence rates of the following events were calculated; treatment initiation, metastasis, death due to prostate cancer and all‐cause mortality, accounting for competing risks. The Cox model of all‐cause mortality and Fine‐Gray sub distribution model to account for competing risks were used to test for racial/ethnic differences in outcomes adjusted for clinical factors.

**Results:**

There were 3925 men in the study, 749 Hispanic, 2415 non‐Hispanic white, 559 non‐Hispanic African American, and 202 non‐Hispanic Asian/Pacific Islander (API). Median follow‐up was 9.3 years. At 19 years, overall cumulative incidence of treatment, metastasis, death due to prostate cancer, and all‐cause mortality was 25.0%, 14.7%, 11.7%, and 67.8%, respectively. In adjusted models compared to non‐Hispanic whites, African Americans had higher rates of treatment (HR = 1.39, 95% CI = 1.15–1.68); they had an increased risk of metastasis beyond 10 years after diagnosis (HR = 4.70, 95% CI = 2.30–9.61); API and Hispanic had lower rates of all‐cause mortality (HR = 0.66, 95% CI = 0.52–0.84, and HR = 0.72, 95% CI = 0.62–0.85, respectively), and API had lower rates of prostate cancer mortality in the first 10 years after diagnosis (HR = 0.29, 95% CI = 0.09–0.90) and elevated risks beyond 10 years (HR = 5.41, 95% CI = 1.39–21.11).

**Conclusions:**

Significant risks of metastasis and prostate cancer mortality exist in untreated men beyond 10 years after diagnosis, but are not equally distributed among racial/ethnic groups.

## Graphical abstract

This retrospective cohort study examined the long‐term clinical outcomes for men within racial/ethnic groups diagnosed with localized prostate cancer who did not undergo initial treatment. We show that significant risks of metastasis and prostate cancer mortality exist in untreated men beyond 10 years after diagnosis, but the risks are not equally distributed among racial/ethnic groups.

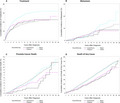

## 1 INTRODUCTION

Prostate cancer is the most common cancer and second most common cause of cancer‐related mortality in men, with an estimated 192,443 men diagnosed with and 30,370 deaths from prostate cancer in 2016.^1^ Approximately 80% of prostate cancers are diagnosed at the localized stage.^2^ Except for a minority that can be lethal, most localized prostate cancers are indolent tumors which would not require treatment,^3^ while treatments such as surgery and radiation are often associated with adverse side effects including incontinence, sexual dysfunction, and reduced quality of life.^3^, ^4^ As a result, an increasing number of men with localized prostate cancer choose surveillance rather than curative treatment.^5^

As men diagnosed with localized cancer are likely to have many years of life remaining, long‐term outcomes of men without curative treatment are important to study.^6^, ^7^, ^8^ Such data can help guide and improve decision‐making among newly diagnosed men. However, available studies have included single‐race cohorts^7^, ^8^, ^9^ or have not directly compared outcomes by race/ethnicity.^10^, ^11^ Those that have made the comparison have generally been in men who received curative treatment at diagnosis^12^, ^13^ or across all treatment modalities.^14^, ^15^, ^16^, ^17^ Many studies rely on SEER cancer registry data,^17^, ^18^ which lacks detailed clinical characteristics such as late treatments and metastasis after diagnosis. The few studies of the natural history of prostate cancer in a racially diverse group of men who were not initially treated with long‐term follow‐up are generally comprised of men diagnosed before the era of PSA screening.^7^, ^8^

It is generally recognized that African American prostate cancer patients have higher mortality from the disease than non‐Hispanic white (NHW) patients,^16^, ^19^ though some studies have suggested that after adjustment for clinical factors, they are at no higher risk of mortality than NHW.^13^, ^20^, ^21^ On the other hand, Asian men have been shown to have lower incidence and lower mortality from prostate cancer.^17^, ^19^, ^22^ Studies of Hispanic men have generally shown them to be at similar risk of prostate cancer mortality to NHW.^15^, ^18^ However, the lack of long‐term data on racial/ethnic groups of untreated men prevents physicians from providing relevant race‐specific information to patients facing treatment decisions.

To address this critical gap in the literature, we assembled a cohort of racially/ethnically diverse men diagnosed with localized prostate cancer from 1997–2007 who went on surveillance in the largest community‐based oncology practice in the US. In this study, we report long‐term clinical outcomes up to 19 years after diagnosis, including treatment initiation, development of metastatic disease, and death due to prostate cancer or other causes overall and within race/ethnicity groups.

## 2 METHODS

### 2.1 Study setting and population

This study was conducted at Kaiser Permanente Southern California (KPSC), an integrated healthcare delivery system that provides comprehensive health services to over 4.5 million racially/ethnically and socioeconomically diverse members who are broadly representative of residents in Southern California.^23^ Men who met the following inclusion criteria were included in the study: (1) newly diagnosed with prostate cancer between January 1, 1997 and December 31, 2007 within KPSC; (2) diagnosed at localized stage (TNM or SEER stage 1 and 2); (3) Gleason score of 7 or less on diagnostic biopsy; and (4) did not receive curative treatment (prostatectomy or radiation therapy) within 1 year of diagnosis. Cancers were identified using a SEER‐affiliated cancer registry. Men were excluded who met the following criteria: (1) tumor grade Gleason 8 or higher at diagnosis; (2) those with unknown race/ethnicity or those who were not of the four main race/ethnic groups (NHW, black, Hispanic, and Asian/Pacific Islander); or (3) those who died, were identified to have metastatic disease or were lost to follow‐up within 1 year of diagnosis. Men in the study cohort were followed until health plan disenrollment, death, or December 31, 2016, whichever came first. The study was reviewed and approved by the KPSC IRB.

### 2.2 Data collection

The main exposure of interest was race/ethnicity, categorized as Hispanic, non‐Hispanic white (NHW), non‐Hispanic African American, and non‐Hispanic Asians/Pacific Islander (API). Other covariates of interest included age at diagnosis, tumor stage and grade, number and percentage of biopsy cores positive for cancer, PSA level and trajectory, and socioeconomic status. Race/ethnicity, age at diagnosis, and Gleason score (beginning in 2003) were identified from the cancer registry, supplemented by the electronic health records. The data quality of the KPSC cancer registry is assured by the SEER standard. Natural language processing of electronic health records, including the pathology report for the diagnostic biopsy, was employed to extract Gleason scores (when not available in cancer registry data), number of biopsy cores taken and number positive for cancer, and percentage of biopsy cores positive for cancer from the pathology report. Serum PSA level at diagnosis was taken as the closest lab value within the 6 months prior to diagnosis. PSA doubling time (PSADT) was calculated for each man using log‐linear regression on all serum PSA values within 2 years prior to the diagnosis date, with a minimum of 2 weeks between the first and last measures. Nearly 20% of men did not have sufficient serum PSA measurements to calculate PSADT, so a missing category was included in analyses. The rate of PSA testing during follow‐up was calculated as the number of PSA tests between diagnosis and the first of treatment initiation, metastasis, loss to follow‐up or death, divided by the duration of that interval in years. The Charlson Comorbidity Index^24^ was calculated based on diagnoses in the year prior to prostate cancer diagnosis. AUA risk stratification into low, intermediate, and high‐risk groups was based on Gleason, PSA level, and stage.^27^ Neighborhood median household income was determined from 2000 US census data for the block group including the patient's home address at the time of diagnosis.

The outcomes of interest included initiation of treatment, metastasis, prostate‐specific mortality, and overall mortality. For initiation of curative treatment, prostatectomy, brachytherapy, external beam radiation therapy, chemotherapy, and immunotherapy were identified using ICD‐9, ICD‐10, and CPT procedure codes in the electronic health records. Suspected metastases were identified using ICD‐9 diagnosis codes for metastatic cancer, natural language processing of radiology reports, serum PSA levels >20 ng/ml, initiation of chemotherapy (any antineoplastic agent) or hormonal therapy (leuprolide), encounters with an oncologist at least 12 months following diagnosis, and death due to prostate cancer. Records of those with suspected metastasis were manually reviewed to determine true metastasis status and date. An experienced urologist (GWC) reviewed all questionable cases and made a final determination on the presence of metastasis. Deaths were identified from clinical records and death certificate linkage with state of California and Social Security Administration records. Prostate cancer mortality was identified as death with the underlying cause coded as prostate cancer on the death certificate.

### 2.3 Statistical analysis

Descriptive statistics were calculated as frequency and percentage for categorical variables and mean, standard deviation, median, and range for continuous factors. Bivariate associations of demographic and clinical factors with race/ethnicity were tested using the chi‐square test or Kruskal‐Wallis test. Time‐to‐event methods were used to evaluate outcomes. Treatment and metastasis were considered competing events, as were death due to prostate cancer and other causes. Unadjusted cumulative incidence rates were estimated using competing risks cumulative incidence methods.^25^ Hazard ratios (HR) for overall survival were estimated using the Cox proportional hazards model, while HRs for other outcomes were estimated using the Fine‐Gray method to account for competing risks. Multivariable models were used to estimate the associations between the outcomes and race/ethnicity adjusted for age, Gleason, Charlson comorbidity index, PSA level at diagnosis, PSADT, stage at diagnosis (2 vs. 1), and neighborhood median household income. Deviations from the proportional hazards assumption were assessed graphically and using correlation with time to event. As deviation from proportionality was observed among API men for prostate cancer mortality and African Americans for metastasis, a term allowing a change in relative risk after 10 years was added to those models. All analyses were conducted in SAS Enterprise Guide version 7.1 statistical software. All tests were two‐sided and *p*‐values <0.05 were taken to indicate statistical significance. The data that support the findings of this study are available from the corresponding author upon reasonable request.

## 3 RESULTS

**Figure 1:**
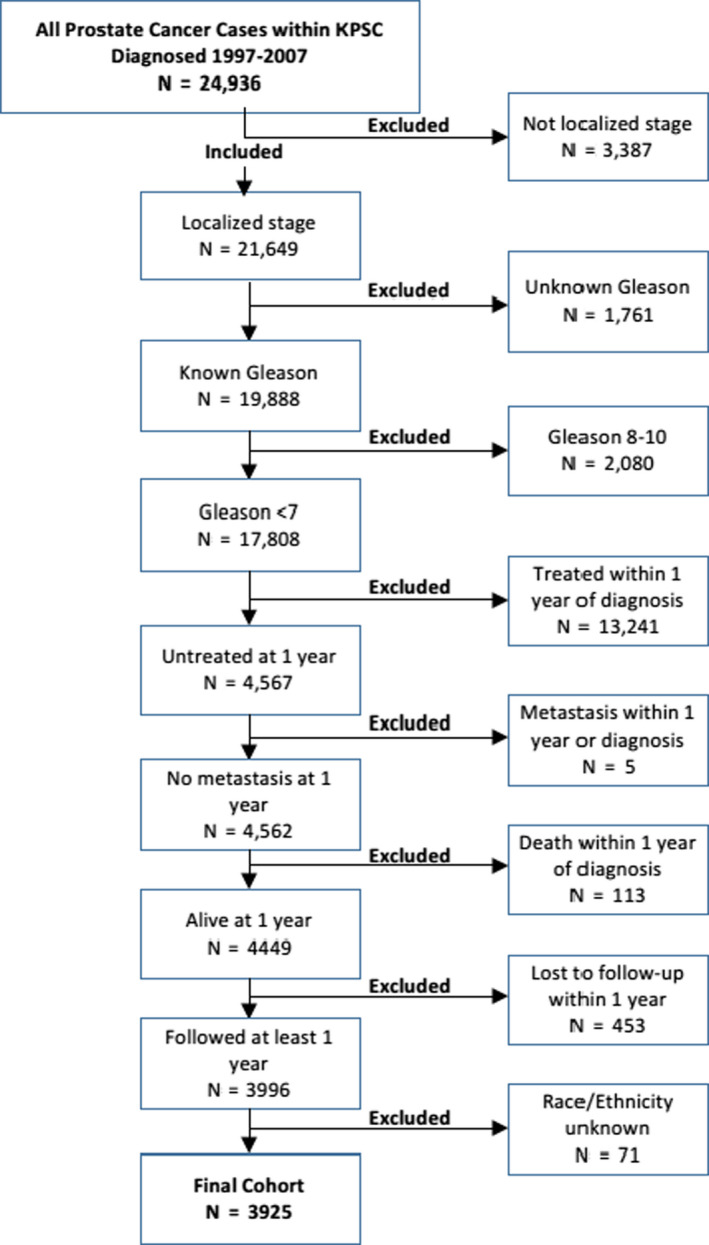
Cohort selection starting with all prostate cancer cases within KPSC diagnosed 1997–2007 and showing the number excluded with not localized stage, unknown Gleason, Gleason 8–10, being treated within 1 year of diagnosis, metastasis within 1 year of diagnosis, death within 1 year of diagnosis, lost to follow‐up within 1 year and race/ethnicity unknown

A total of 24,936 men were diagnosed with localized prostate cancer between 1997 and 2007, with 3925 included in the study cohort after applying the inclusion and exclusion criteria (Figure 1).

There were several notable differences seen across race/ethnicity groups (Table 1). NHW tended to be older at diagnosis (mean 70.0 years) and African Americans tended to be younger (mean 66.1 years). African Americans were most likely to have a Gleason grade >6 (33.1%), while Hispanics were least likely (26.5%). Diagnosis with a PSA level of 10.0 ng/ml or higher was more likely in API (35.5%) and African Americans (33.7%) than in NHW (27.4%) and Hispanics (29.0%), while African Americans were more likely to have a PSA doubling time of <3 years (35.6%) and API was least likely (23.8%). Hispanic (57.6%) and NHW (56.6%) were most likely to have AUA low‐risk disease, while API had the highest rate of intermediate‐risk tumors (44.1%) and African Americans had the highest rate of high‐risk tumors (10.4%) There were no statistically significant differences in the rate of PSA testing during follow‐up, Charlson scores, the number of positive biopsy cores or the greatest percentage of cancer in a biopsy core. The median neighborhood household income was lowest in African Americans (mean $44,036) and highest in NHW (mean $57,667) and API ($56,683).

**Table 1:** Description of cohort by race/ethnicity

	Non‐Hispanic white (*n* = 2415)	African American (*n* = 749)	Hispanic (*n* = 559)	Asian/PI (*n* = 202)	Total (*n* = 3925)	*p*‐value
Stage						0.35
1	141 (5.8%)	40 (5.3%)	23 (4.1%)	16 (7.9%)	220 (5.6%)	
2	2244 (92.9%)	703 (93.9%)	531 (95%)	185 (91.6%)	3663 (93.3%)	
Unknown	30 (1.2%)	6 (0.8%)	5 (0.9%)	1 (0.5%)	42 (1.1%)	
Age at diagnosis						<0.0001
Mean (SD)	70.0 (9.3)	66.1 (9.5)	67.6 (9.3)	68.2 (8.3)	68.8 (9.4)	
Range	(40.1–96.4)	(40.9–90.4)	(40.7–95.7)	(46.0–87.6)	(40.1–96.4)	
Gleason						0.03
≤6	1738 (72.0%)	501 (66.9%)	411 (73.5%)	141 (69.8%)	2791 (71.1%)	
7	677 (28.0%)	248 (33.1%)	148 (26.5%)	61 (30.2%)	1134 (28.9%)	
PSA at diagnosis						0.002
0–3.99	340 (14.8%)	83 (11.6%)	88 (16.6%)	18 (9.5%)	529 (14.2%)	
4.0–9.99	1331 (57.8%)	390 (54.6%)	288 (54.4%)	104 (55.0%)	2113 (56.6%)	
10–19.99	443 (19.2%)	166 (23.2%)	114 (21.6%)	54 (28.6%)	777 (20.8%)	
20+	190 (8.2%)	75 (10.5%)	39 (7.4%)	13 (6.9%)	317 (8.5%)	
Missing	111	35	30	13	189	
AUA risk stratification						0.001
Low	1368 (56.6%)	362 (48.3%)	322 (57.6%)	100 (49.5%)	2152 (54.8%)	
Intermediate	852 (35.3%)	309 (41.3%)	197 (35.2%)	89 (44.1%)	1447 (36.9%)	
High	195 (8.1%)	78 (10.4%)	40 (7.2%)	13 (6.4%)	326 (8.3%)	
PSA doubling time						0.01
Negative or stable	841 (41.0%)	201 (33.0%)	178 (39.3%)	74 (43.0%)	1294 (39.4%)	
<3 years	593 (28.9%)	217 (35.6%)	138 (30.5%)	41 (23.8%)	989 (30.1%)	
≥3 years	619 (30.2%)	192 (31.5%)	137 (30.2%)	57 (33.1%)	1005 (30.6%)	
Missing	362	139	106	30	637	
Charlson score						0.59
0	1571 (65.1%)	469 (62.6%)	349 (62.4%)	129 (63.9%)	2518 (64.2%)	
1	416 (17.2%)	120 (16.0%)	101 (18.1%)	37 (18.3%)	674 (17.2%)	
2	225 (9.3%)	83 (11.1%)	63 (11.3%)	18 (8.9%)	389 (9.9%)	
3+	203 (8.4%)	77 (10.3%)	46 (8.2%)	18 (8.9%)	344 (8.8%)	
Years membership before dx						<0.0001
Mean (SD)	16.0 (12.5)	17.8 (12.7)	12.6 (11.3)	15.1 (10.8)	15.8 (12.4)	
Follow‐up after diagnosis (years)						0.02
Mean (SD)	8.7 (4.7)	9.1 (4.8)	8.7 (4.8)	9.8 (4.9)	8.8 (4.7)	
Follow‐up PSA tests/year						0.19
Mean (SD)	4.6 (5.4)	5.6 (7.2)	5.5 (6.9)	5.1 (6.4)	4.9 (6.1)	
Highest biopsy core percent positive						0.94
*N*	1001	260	224	83	1568	
Mean (SD)	27.9 (26.3)	28.5 (26.4)	25.6 (24.0)	28.1 (26.2)	27.7 (26.0)	
Missing *N*=						
Number of positive biopsy cores						0.6184
*N*	776	241	183	68	1268	
Mean (SD)	2.7 (2.2)	3.0 (2.6)	2.6 (2.0)	2.6 (2.0)	2.7 (2.3)	
Missing *N*=						
Neighborhood household median income ($)						<0.0001
Mean (SD)	57,666.7 (24,286.1)	44,035.8 (20,491.2)	46,918.1 (19,639.9)	56,682.9 (23,759.9)	53,494.5 (23,702.7)	

Abbreviations: dx, diagnosis; PI, Pacific Islander; PSA, prostate‐specific antigen; SD, standard deviation.

**Figure 2:**
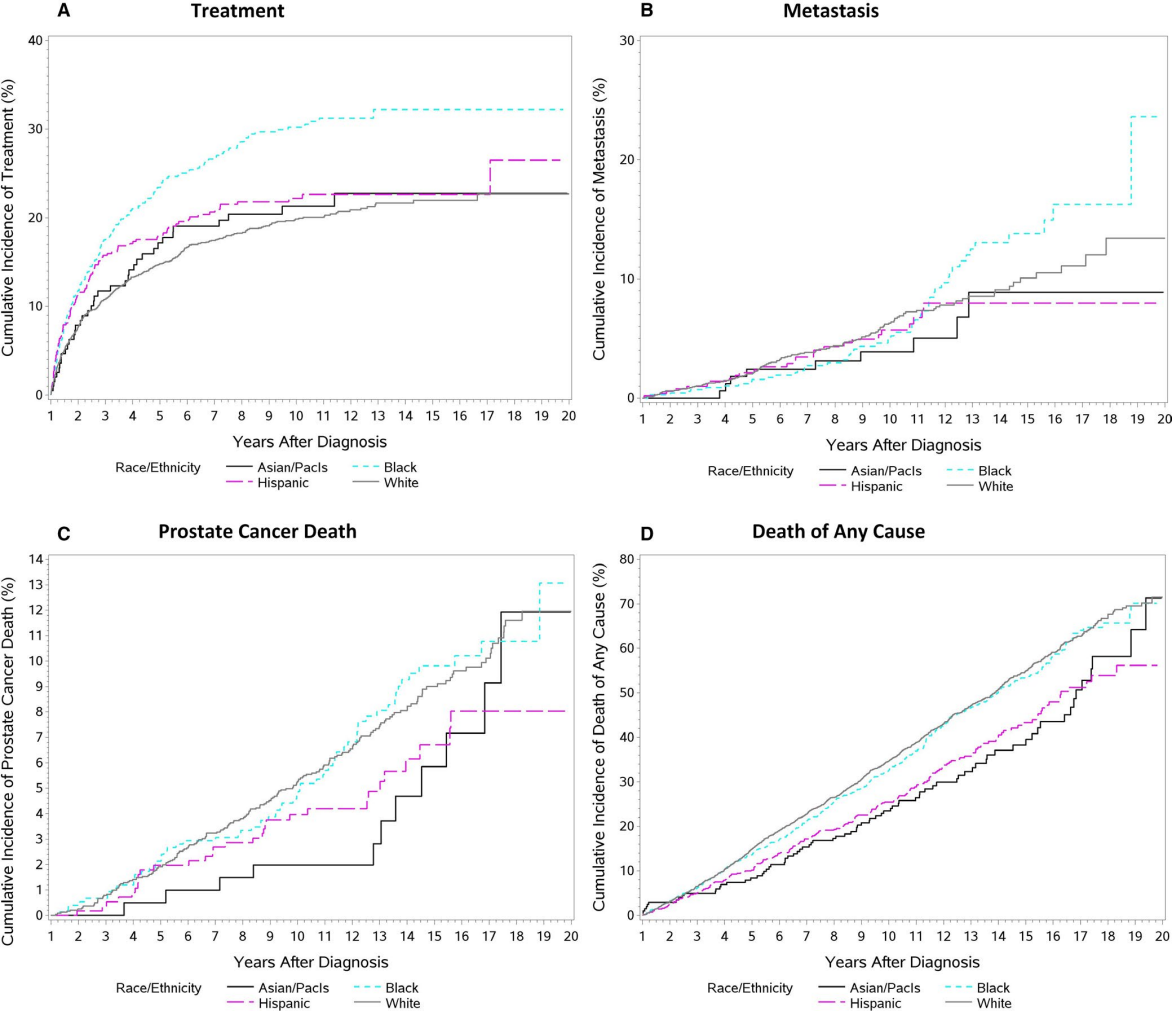
Cumulative incidence of long‐term clinical outcomes following prostate cancer diagnosis by race/ethnicity: (A) treatment; (B) metastasis; (C) prostate cancer death; (D) death of any cause

The overall cumulative incidence of treatment in the absence of metastasis was 17.1%, 22.3%, 24.1%, and 25.0% at 5, 10, 15, and 19 years after diagnosis, respectively. Incidence of treatment was higher in African Americans (32.2% at 19 years) and Hispanic (26.5%) than in API and NHW (both 22.7%, Table 2, Figure 2(A)). In a multivariable Fine‐Gray model accounting for the competing risk of metastasis, African Americans were significantly more likely to receive treatment (HR = 1.39, 95% CI = 1.15–1.68), while Hispanics (HR = 1.18, CI = 0.93–1.49) and API (HR = 1.04, CI = 0.74–1.46) did not significantly differ from NHW (Table 3). Younger ages, lower Charlson scores, lower AUA risk strata, diagnosis at stage 2, and longer PSA doubling time were associated with higher rates of treatment.

**Table 2:** Cumulative incidence estimates, accounting for competing risks

#	Non‐Hispanic white	African American	Hispanic	Asian/PI	Overall
Cumulative incidence of treatment					
5 years	14.8%	23.4%	18.0%	17.2%	17.1%
10 years	19.9%	30.2%	22.1%	21.3%	22.3%
15 years	22.0%	32.2%	22.7%	22.7%	24.1%
19 years	22.7%	32.2%	26.5%	22.7%	25.0%
Cumulative incidence of metastasis					
5 years	2.1%	1.6%	2.1%	2.4%	2.0%
10 years	6.3%	4.9%	5.7%	3.9%	5.8%
15 years	10.1%	13.8%	8.0%	8.9%	10.6%
19 years	13.4%	23.6%	8.0%	8.9%	14.7%
Cumulative incidence of death due to prostate cancer					
5 years	1.9%	2.1%	2.0%	0.5%	1.9%
10 years	5.3%	4.7%	4.0%	2.0%	4.8%
15 years	9.0%	9.8%	6.7%	5.9%	8.7%
19 years	12.0%	13.1%	8.0%	11.9%	11.7%
Cumulative incidence of death of any cause					
5 years	14.9%	13.6%	10.2%	8.4%	13.7%
10 years	34.7%	32.7%	25.5%	23.7%	32.4%
15 years	55.0%	53.4%	43.3%	38.3%	52.2%
19 years	69.6%	70.1%	56.2%	64.2%	67.8%

Abbreviation: PI, Pacific Islander.

**Table 3:** Model parameter estimates for treatment, metastasis, all‐cause mortality, and prostate cancer mortality. Fine‐Gray competing risks model estimates except all‐cause mortality which does not have a competing risk

Parameter	Treatment	Metastasis	All‐cause mortality	Prostate cancer‐specific mortality
	Hazard ratio (95% CI)	Hazard ratio (95% CI)	Hazard ratio (95% CI)	Hazard ratio (95% CI)
Non‐Hispanic white	1.0 (ref)	1.0 (ref)	1.0 (ref)	1.0 (ref)
Asian/Pacific Islander	1.04 (0.74–1.46)	0.57 (0.25–1.28)	0.66 (0.52–0.84)	–
Asian/PI, first 10 years of follow‐up	–	–		0.29 (0.09–0.90)
Asian/PI, beyond 10 years of follow‐up	–	–		5.41 (1.39–21.11)
Black	1.39 (1.15–1.68)	–	1.10 (0.96–1.25)	1.06 (0.77–1.46)
Black, first 10 years of follow‐up	–	0.68 (0.41–1.12)		–
Black, beyond 10 years of follow‐up	–	4.70 (2.30–9.61)		–
Hispanic	1.18 (0.93–1.49)	0.88 (0.55–1.40)	0.72 (0.62–0.85)	0.73 (0.48–1.10)
Gleason < 6	1.0 (ref)	1.0 (ref)	1.0 (ref)	1.0 (ref)
Gleason 7	0.76 (0.59–0.98	1.97 (1.37–2.85)	1.12 (0.98–1.29)	1.76 (1.28–2.43)
AUA low risk	1.0 (ref)	1.0 (ref)	1.0 (ref)	1.0 (ref)
AUA intermediate risk	1.13 (0.88–1.46)	1.19 (0.75–1.88)	1.23 (1.06–1.43)	1.05 (0.72–1.53)
AUA high risk	0.80 (0.47–1.36)	1.69 (0.84–3.42)	1.57 (1.21–2.03)	1.15 (0.60–2.22)
Age < 55	1.0 (ref)	1.0 (ref)	1.0 (ref)	1.0 (ref)
Age 55–64	0.83 (0.65–1.05)	1.84 (0.64–5.31)	1.91 (1.35–2.72)	1.27 (0.60–2.71)
Age 65–74	0.46 (0.36–0.59)	2.61 (0.94–7.29)	3.99 (2.84–5.60)	2.00 (0.96–4.16)
Age 75+	0.18 (0.13–0.25)	4.84 (1.74–13.50)	7.82 (5.55–11.02)	3.59 (1.72–7.48)
Stage 2 vs 1	2.00 (1.40–2.85)	1.14 (0.69–1.86)	0.82 (0.70–0.96)	1.15 (0.77–1.73)
PSA at diagnosis (log‐2 scale)	0.98 (0.90–1.08)	1.41 (1.16–1.72)	1.09 (1.01–1.16)	1.26 (1.06–1.51)
PSA doubling time negative or stable	1.0 (ref)	1.0 (ref)	1.0 (ref)	1.0 (ref)
PSA doubling time < 3 years	1.15 (0.94–1.41)	1.56 (1.06–2.29)	0.96 (0.84–1.08)	1.18 (0.86–1.61)
PSA doubling time ≥ 3 years	1.29 (1.06–1.58)	1.30 (0.88–1.94)	0.85 (0.75–0.96)	1.06 (0.77–1.46)
PSA doubling time unknown	1.15 (0.90–1.48)	1.66 (1.05–2.63)	1.11 (0.95–1.29)	1.33 (0.92–1.90)
Charlson score 0	1.0 (ref)	1.0 (ref)	1.0 (ref)	1.0 (ref)
Charlson score 1	1.07 (0.87–1.31)	0.84 (0.56–1.25)	1.40 (1.24–1.58)	0.67 (0.47–0.94)
Charlson score 2	0.72 (0.53–0.98)	0.77 (0.45–1.30)	1.67 (1.42–1.95)	0.64 (0.40–1.01)
Charlson score 3+	0.79 (0.57–1.10)	0.96 (0.53–1.74)	2.32 (1.96–2.75)	0.79 (0.50–1.24)
Neighborhood median household income (per $10,000)	1.01 (0.98–1.04)	0.95 (0.88–1.03)	0.96 (0.94–0.98)	0.97 (0.91–1.03)

Abbreviations: CI, confidence interval; PI, Pacific Islander; PSA: prostate‐specific antigen.

The overall cumulative incidence of treatment‐naïve metastasis was 2.0%, 5.8%, 10.6%, and 14.7% at 5, 10, 15, and 19 years, respectively. Incidence of metastasis was highest in African Americans (23.6% at 19 years), lower in NHW (13.4%), and lowest in API (8.9%) and Hispanic men (8.0%, Table 2, Figure 2(B)). However, the increased rate of metastasis in African Americans was not consistent across the duration of follow‐up; their observed rate was lower at 10 years (4.9%) than in Hispanics (5.7%) and NHW (6.3%), after which their metastasis rate increased notably relative to other groups. In the adjusted model, Hispanics were at similar risk to NHW (HR = 0.88, CI = 0.55–1.40) and API was at somewhat reduced risk (HR = 0.57, CI = 0.25–1.28). African Americans were at a similar risk compared to NHW for the first 10 years following diagnosis (HR = 0.68, CI = 0.41–1.12), but at increased risk after 10 years (HR = 4.70, CI = 2.30–9.61). Higher Gleason scores, higher AUA risk strata, older ages, higher PSA level at diagnosis, and PSA doubling time <3 years were significantly associated with increased rates of metastasis.

The overall cumulative incidence of prostate cancer mortality was 1.9%, 4.8%, 8.7%, and 11.7% at 5, 10, 15, and 19 years after diagnosis, respectively, with the rates highest in African Americans (13.1% at 19 years), lower in NHW (12.0%) and API (11.9%) and lowest in Hispanics (8.0%, Table 2, Figure 2(C)). The rate in API men was lowest at 10 years and then increased relative to the other groups. The adjusted Fine‐Gray sub distribution model estimates the hazard ratio for API relative to NHW to be lower in the first 10 years of follow‐up (HR = 0.29, CI = 0.09–0.90) and higher after 10 years (HR = 5.41, CI = 1.39–21.11). African Americans were not at significantly higher risk of prostate cancer mortality than NHW (HR = 1.06, CI = 0.77–1.46), while Hispanics were not at significantly reduced risk (HR = 0.73, CI = 0.48–1.10). Older age at diagnosis, higher Gleason score, and PSA level were associated with increased risk of prostate cancer mortality.

The overall cumulative incidence of death from any cause was 13.7%, 32.4%, 52.2%, and 67.8% at 5, 10, 15, and 19 years, respectively. Mortality rates were highest in African Americans (70.1%) and NHW (69.6%), lower in API (64.2%), and lowest in Hispanics (56.2%). In an adjusted Cox model, API (HR = 0.66, CI = 0.52–0.84) and Hispanics (HR = 0.72, CI = 0.62–0.85) had reduced risk relative to NHW men. African Americans were not at statistically significantly increased risk (HR = 1.10, CI = 0.96–1.25). Men diagnosed at older ages, with higher Charlson, Gleason, AUA risk strata and PSA, stage 1, and lower neighborhood median income levels were at significantly increased risk of all‐cause mortality.

Stratified analysis by AUA risk group showed similar associations of outcomes with race/ethnicity and other clinical factors (Supplemental Table). Higher‐risk patients had higher rates of metastasis, prostate cancer mortality, and all‐cause mortality, while they had lower rates of treatment initiation (Supplemental Figure).

## 4 DISCUSSION

This large, multi‐ethnic cohort provides insights into the long‐term outcomes of treatment, metastasis, and mortality. Similar to what others have shown, it indicates that a significant risk of metastasis and prostate cancer mortality exists beyond 10 years after diagnosis. There are significant differences in outcomes among the different race/ethnicity groups in the study, particularly lower rates of metastasis and all‐cause mortality in API and Hispanic men. The increasing risks of metastasis beyond 10 years in African Americans men and prostate cancer mortality in API men appear to be novel findings that warrant further investigation.

One surprising finding was that African Americans men were more likely to receive treatment in the absence of confirmed metastatic disease. During the first 10 years of follow‐up, their rate of metastasis was similar to NHW and Hispanic men, after which it increased significantly. Several studies have reported metastasis rates that are similar between NHW and African Americans,^12^, ^13^ though their follow‐up was limited to 10–12 years. Further study is needed to confirm this finding. Shaver reported lower‐intensity monitoring in African Americans on watchful waiting, which could result in delayed identification of cancer progression. However, in our cohort, African American men had the highest rate of PSA monitoring during follow‐up, averaging over 5.5 tests/year.

Hispanic men were similar to NHW in terms of rates of treatment and metastasis. Their rates of prostate cancer mortality were slightly but not statistically significantly improved compared to NHW men. Tyson reported similar findings, with 10‐year cancer‐specific survival rates of 87.5% in untreated Hispanic men and 86.3% in untreated NHW. Hispanic men were observed to have better all‐cause survival than NHW, with an estimated 25% reduction in mortality. Tyson reported no significant overall mortality benefit after adjusting for clinical and treatment factors (HR = 0.97, 95% CI = 0.94–1.01), while in our cohort the mortality benefit remained after adjustment.

API men have long been known to have a lower risk of developing prostate cancer compared to NHW and African Americans. In our study, they were just as likely to receive treatment in the absence of confirmed metastasis as NHW. Their rate of treatment‐naïve metastasis was not statistically significantly lower than NHW, though the point estimate suggests a lower rate. Man and colleagues reported a similar finding for biochemical failure in men treated with radiotherapy, while Cohen reported no difference in the rate of disease recurrence. Interestingly, while their rate of prostate cancer‐specific mortality is significantly lower than other racial/ethnic groups for the first 10 years following diagnosis, the opposite was observed beyond 10 years, to the point that at 19 years their cumulative incidence of mortality equals that of NHW. This finding appears to be novel and warrants additional study. Most studies have shown API men to be at lower risk of adverse prostate cancer outcomes compared to NHW, though Man reported no significant advantage in biochemical failure or cause‐specific survival following radiotherapy and Cohen reported no significant advantage in disease recurrence, though neither study had significant follow‐up beyond 10 years. API men were observed to have a significantly lower all‐cause mortality rate than NHW. This finding is consistent with the literature, with Holmes reporting a very similar 37% reduction in risk in an adjusted model.

This study has several potential limitations which should be acknowledged. First, the cohort was defined by not receiving treatment within 12 months of diagnosis, rather than identifying those who specifically selected active surveillance or watchful waiting. The retrospective nature of the study made this impossible to determine. A sensitivity analysis was conducted using 6 months to determine absence of initial treatment. None of the primary findings were significantly altered using this cohort, though we did see a larger number of Hispanic men treated between 6 and 12 months. This complements the findings of Lichtensztajn, who found that Hispanic men were less likely to receive treatment compared with NHW^26^ in the first 6 months. This suggests that Hispanic men are simply more likely to delay their initial treatment beyond 6 months; hence using 12 months to define initial treatment would be more appropriate.

Second, our cohort included a significant percentage of men with intermediate risk and a small number of high‐risk prostate cancers for whom therapy is generally recommended unless life expectancy is <5 years.^27^ However, we conducted a stratified analyses by AUA risk groups, which showed that while absolute rates differed, the associations with race/ethnicity and other factors were similar across risk groups. Including these men in our study may provide useful information on potential outcomes in the absence of treatment for men at higher risk.

Finally, most data were collected retrospectively from the medical records and may not be as accurate as prospectively collected data. We took several steps to ensure the quality and completeness of the data. While some metastasis cases may have been missed due to a lack of clear documentation, a broad search of potential indicators and chart reviews were used to identify metastases. An experienced urologist (GWC) reviewed all questionable cases and made a final determination of metastasis.

This study has several important strengths, including the large, multi‐ethnic cohort, the complete capture of treatments and outcomes within a closed healthcare system, and the long‐term follow‐up of these men.

These data highlight the fact that there remains significant risk of metastasis and mortality beyond 10 years in men with untreated localized prostate cancer, but this risk is not consistent across race/ethnicity groups. Despite observed differences in outcomes, most men in all studied racial/ethnic groups had favorable outcomes, which suggests that race should not be a disqualifying factor in choosing active surveillance among men with low‐risk prostate cancer. Knowledge of these risks and their differences should help clinicians in their discussions with patients to better weigh risks and benefits of expectant management versus immediate treatment.

## CONFLICT OF INTEREST

All authors, no conflicts of interest.

## AUTHOR CONTRIBUTIONS

Jeff M. Slezak collected, assembled, analyzed, and interpreted the data and wrote the paper. Chun R. Chao designed the research, obtained funding, supervised the study, analyzed and interpreted the data, and contributed to the writing of the paper. Kimberly L. Cannavale collected and assembled the data and contributed to the writing of the paper. Stephen K. Van Den Eeden, Gary W. Chien, and Steven J. Jacobsen interpreted the findings and critically contributed to the writing of the paper.

## Supporting information

Fig S1‐S3

Table S1‐S4

## Data Availability

The data that support the findings of this study are available from the corresponding author upon reasonable request.
