# Supplementary material for: Long‐term follow‐up of a racially and ethnically diverse population of men with localized prostate cancer who did not undergo initial active treatment
Source: Cancer Med. 2020 Sep 23;9(22):8530–9. doi: 10.1002/cam4.3471 (PMC7666755; doi:10.1002/cam4.3471)

Supplemental Figure 1. Cumulative Incidence of Long-term Clinical Outcomes following Prostate Cancer Diagnosis by Race/Ethnicity in Low-Risk Patients
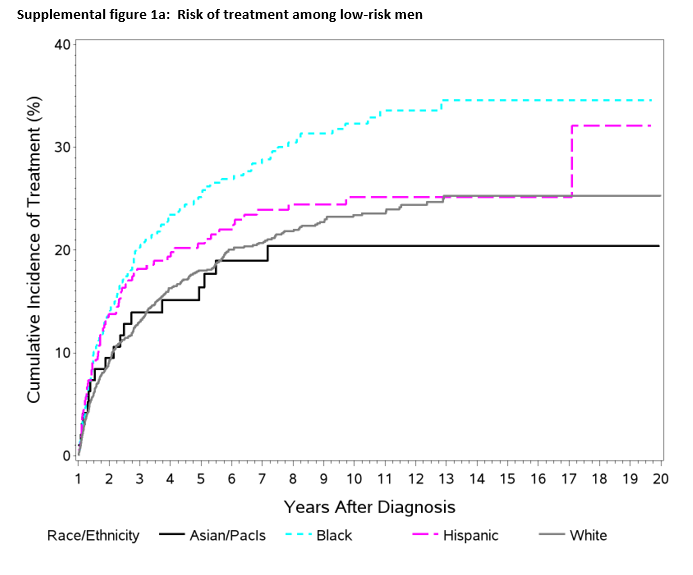

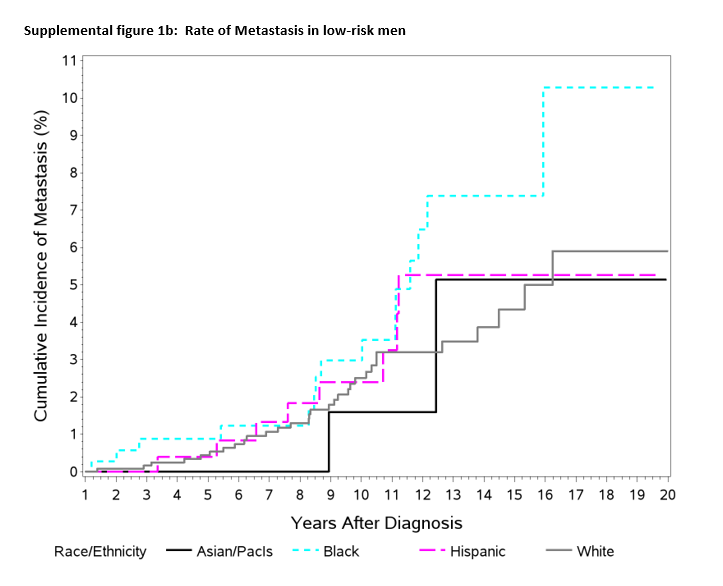

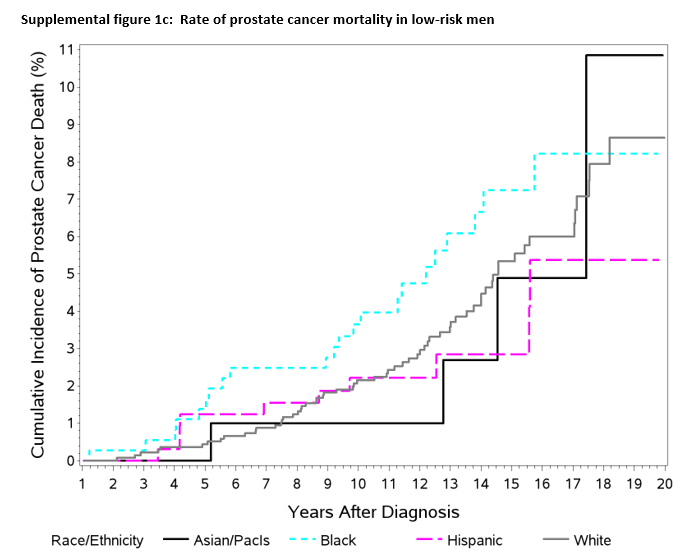

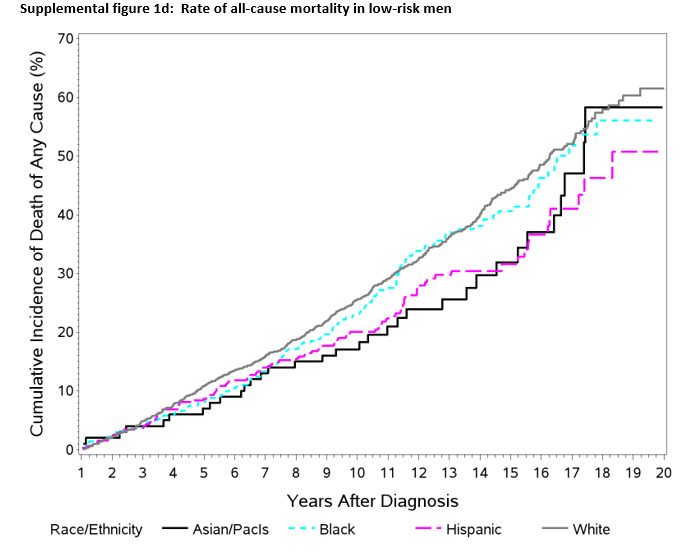


Supplemental Figure 2. Cumulative Incidence of Long-term Clinical Outcomes following Prostate Cancer Diagnosis by Race/Ethnicity in Intermediate-Risk Patients


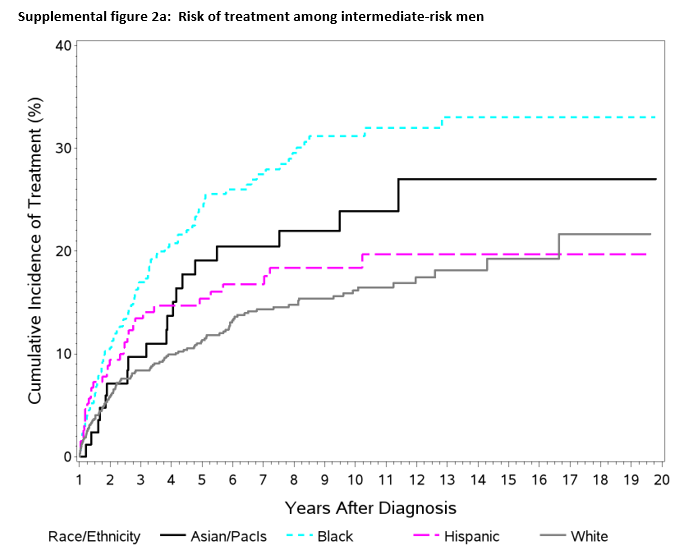

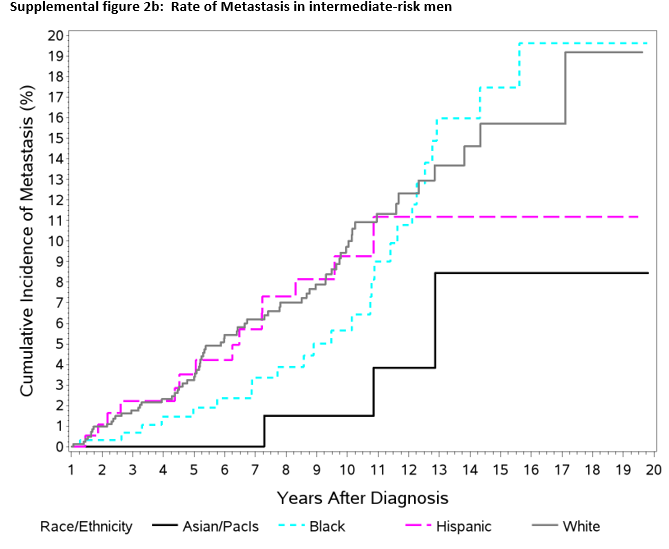

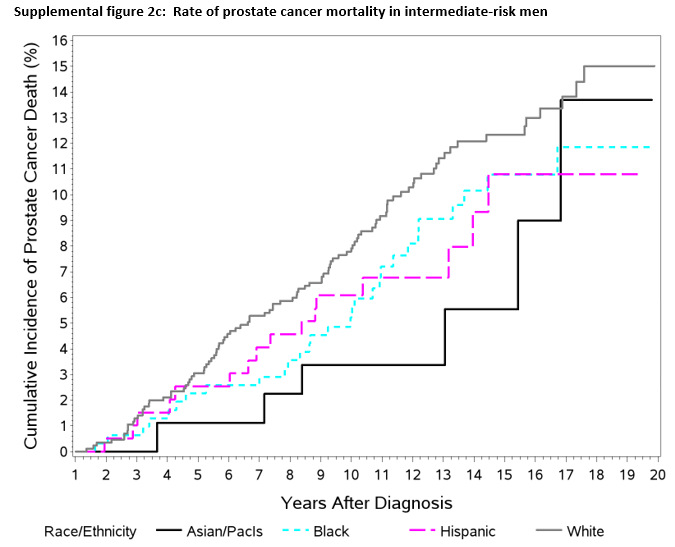

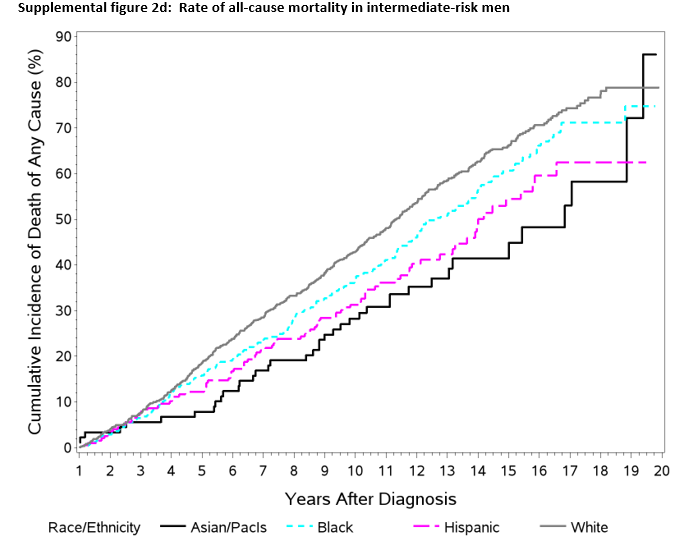


Supplemental Figure 3. Cumulative Incidence of Long-term Clinical Outcomes following Prostate Cancer Diagnosis by Race/Ethnicity in High-Risk Patients


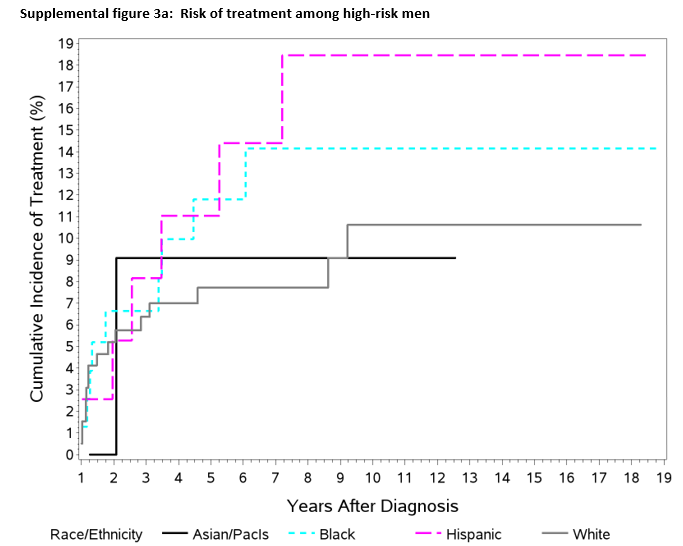

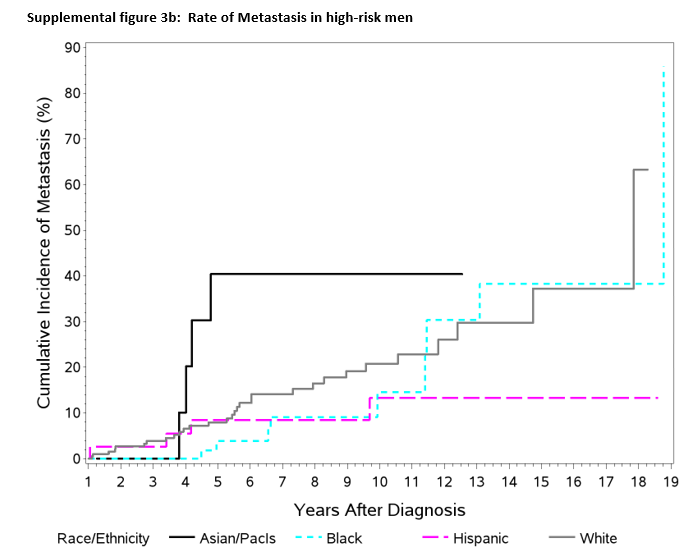


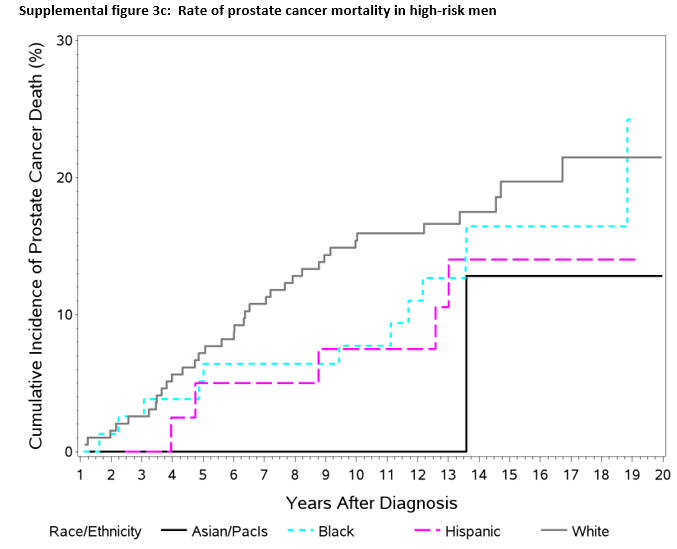

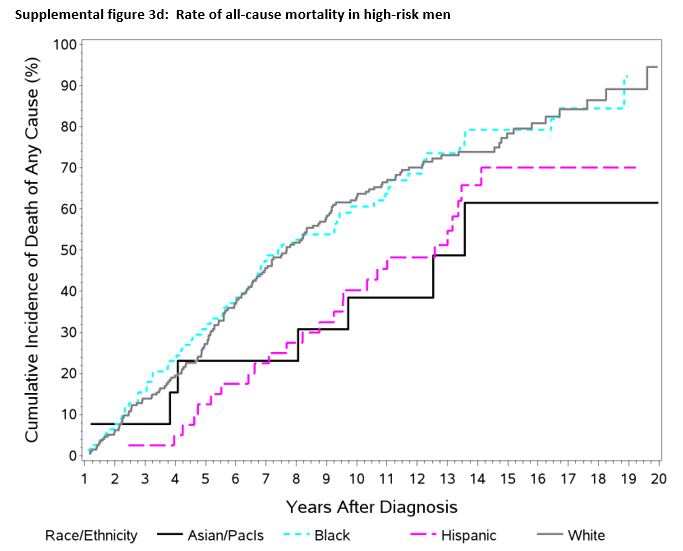

Supplement: Supplementary file 1 — Fig S1‐S3 [file CAM4-9-8530-s001.docx]
