# Supplementary material for: Long‐term follow‐up of a racially and ethnically diverse population of men with localized prostate cancer who did not undergo initial active treatment
Source: Cancer Med. 2020 Sep 23;9(22):8530–9. doi: 10.1002/cam4.3471 (PMC7666755; doi:10.1002/cam4.3471)
Supplement: Supplementary file 2 — Table S1‐S4 [file CAM4-9-8530-s002.docx]

**Supplemental Table 1. Adjusted Cox model for treatment initiation by AUA risk categories (Fine-Gray competing risks model estimates).**

|  |  | **All (n=3925)** | **Low risk (n=2152)** | **Intermediate risk (n=1447)** | **High risk (n=326)** |
| --- | --- | --- | --- | --- | --- |
| **Variable** | **Level** | **Hazard Ratio (95% CI)** | | |  |
| Race/ethnicity | Asian | 1.04 ( 0.74- 1.46) | 0.82 ( 0.50- 1.37) | 1.31 ( 0.80- 2.15) | No modeling was performed due to small number |
|  | Black | 1.39 ( 1.15- 1.68) | 1.28 ( 0.99- 1.64) | 1.61 ( 1.17- 2.21) |  |
|  | Hispanic | 1.18 ( 0.93- 1.49) | 1.19 ( 0.89- 1.60) | 1.18 ( 0.76- 1.83) |  |
| Gleason | 7 vs 6 | 0.76 ( 0.59- 0.98) | N/A | 0.75 ( 0.56- 1.01) |  |
| AUA risk strata | Intermediate vs low | 1.13 ( 0.88- 1.46) | N/A | N/A |  |
|  | High vs low | 0.80 ( 0.47- 1.36) | N/A | N/A |  |
| Age at diagnosis | 55-64 vs <55 | 0.83 ( 0.65- 1.05) | 0.76 ( 0.58- 1.01) | 0.92 ( 0.57- 1.47) |  |
|  | 65-74 vs <55 | 0.46 ( 0.36- 0.59) | 0.38 ( 0.28- 0.51) | 0.58 ( 0.36- 0.93) |  |
|  | 75+ vs <55 | 0.18 ( 0.13- 0.25) | 0.16 ( 0.10- 0.25) | 0.23 ( 0.13- 0.40) |  |
| Stage | 2 vs 1 | 2.00 ( 1.40- 2.85) | 2.34 ( 1.42- 3.84) | 1.28 ( 0.74- 2.20) |  |
| PSA at diagnosis | log-2 scale | 0.98 ( 0.90- 1.08) | 1.20 ( 1.05- 1.37) | 0.79 ( 0.68- 0.92) |  |
| PSA doubling time | <3 years | 1.15 ( 0.94- 1.41) | 1.02 ( 0.78- 1.34) | 1.41 ( 0.99- 2.00) |  |
|  | >=3 years | 1.29 ( 1.06- 1.58) | 1.28 ( 1.00- 1.65) | 1.19 ( 0.82- 1.72) |  |
|  | Unknown | 1.15 ( 0.90- 1.48) | 1.10 ( 0.79- 1.54) | 1.28 ( 0.86- 1.92) |  |
| Charlson score | 1 vs 0 | 1.07 ( 0.87- 1.31) | 1.05 ( 0.80- 1.38) | 1.11 ( 0.79- 1.56) |  |
|  | 2 vs 0 | 0.72 ( 0.53- 0.98) | 0.54 ( 0.34- 0.85) | 0.89 ( 0.56- 1.43) |  |
|  | 3+ vs 0 | 0.79 ( 0.57- 1.10) | 0.99 ( 0.66- 1.49) | 0.43 ( 0.21- 0.86) |  |
| Household median income | per $10,000 | 1.01 ( 0.98- 1.04) | 1.00 ( 0.95- 1.04) | 1.03 ( 0.98- 1.08) |  |

**Supplemental Table 2. Adjusted Cox model for metastasis by AUA risk categories (Fine-Gray competing risks model estimates).**

|  |  | **All (n=3925)** | **Low risk (n=2152)** | **Intermediate risk (n=1447)** | **High risk (n=326)** |
| --- | --- | --- | --- | --- | --- |
| **Variable** | **Level** | **Hazard Ratio (95% CI)** | | |  |
| Race/ethnicity | Asian | 0.57 ( 0.25- 1.28) | 0.47 ( 0.07- 3.34) | 0.32 ( 0.08- 1.30) | No modeling was performed due to small number |
|  | Black first 10 yrs | 0.68 ( 0.41- 1.12) | 1.49 ( 0.60- 3.70) | 0.64 ( 0.30- 1.34) |  |
|  | Black after 10 yrs | 4.70 ( 2.30- 9.61) | 2.68 ( 0.82- 8.73) | 6.01 ( 2.13-16.99) |  |
|  | Hispanic | 0.88 ( 0.55- 1.40) | 1.34 ( 0.56- 3.24) | 0.94 ( 0.50- 1.76) |  |
| Gleason | 7 vs 6 | 1.97 ( 1.37- 2.85) | N/A | 2.03 ( 1.27- 3.26) |  |
| AUA risk strata | Intermediate vs low | 1.19 ( 0.75- 1.88) | N/A | N/A |  |
|  | High vs low | 1.69 ( 0.84- 3.42) | N/A | N/A |  |
| Age at diagnosis | 55-64 vs <55 | 1.84 ( 0.64- 5.31) | 1.35 ( 0.37- 4.90) | 1.57 ( 0.20-12.53) |  |
|  | 65-74 vs <55 | 2.61 ( 0.94- 7.29) | 1.91 ( 0.53- 6.90) | 3.58 ( 0.50-25.66) |  |
|  | 75+ vs <55 | 4.84 ( 1.74-13.50) | 4.22 ( 1.11-16.06) | 6.24 ( 0.85-45.55) |  |
| Stage | 2 vs 1 | 1.14 ( 0.69- 1.86) | 0.62 ( 0.29- 1.32) | 1.34 ( 0.64- 2.80) |  |
| PSA at diagnosis | log-2 scale | 1.41 ( 1.16- 1.72) | 1.18 ( 0.80- 1.72) | 1.73 ( 1.18- 2.53) |  |
| PSA doubling time | <3 years | 1.56 ( 1.06- 2.29) | 1.85 ( 0.80- 4.30) | 1.14 ( 0.65- 2.00) |  |
|  | >=3 years | 1.30 ( 0.88- 1.94) | 1.51 ( 0.65- 3.50) | 1.19 ( 0.68- 2.07) |  |
|  | Unknown | 1.66 ( 1.05- 2.63) | 2.81 ( 1.14- 6.92) | 1.29 ( 0.69- 2.42) |  |
| Charlson score | 1 vs 0 | 0.84 ( 0.56- 1.25) | 0.81 ( 0.37- 1.78) | 0.93 ( 0.54- 1.58) |  |
|  | 2 vs 0 | 0.77 ( 0.45- 1.30) | 0.39 ( 0.09- 1.66) | 1.08 ( 0.56- 2.09) |  |
|  | 3+ vs 0 | 0.96 ( 0.53- 1.74) | 0.54 ( 0.13- 2.33) | 1.26 ( 0.56- 2.86) |  |
| Household median income | per $10,000 | 0.95 ( 0.88- 1.03) | 0.98 ( 0.86- 1.11) | 1.01 ( 0.90- 1.13) |  |

**Supplemental** **Table 3. Adjusted Cox model for all-cause mortality by AUA risk categories.**

|  |  | **All (n=3925)** | **Low risk (n=2152)** | **Intermediate risk (n=1447)** | **High risk (n=326)** |
| --- | --- | --- | --- | --- | --- |
| **Variable** | **Level** | **Hazard Ratio (95% CI)** | | |  |
| Race/ethnicity | Asian | 0.66 ( 0.52- 0.84) | 0.79 ( 0.55- 1.13) | 0.65 ( 0.48- 0.89) | No modeling was performed due to small number |
|  | Black | 1.10 ( 0.96- 1.25) | 1.04 ( 0.84- 1.28) | 1.09 ( 0.90- 1.32) |  |
|  | Hispanic | 0.72 ( 0.62- 0.85) | 0.76 ( 0.59- 0.97) | 0.69 ( 0.54- 0.88) |  |
| Gleason | 7 vs 6 | 1.12 ( 0.98- 1.29) | N/A | 1.23 ( 1.04- 1.46) |  |
| AUA risk strata | Intermediate vs low | 1.23 ( 1.06- 1.43) | N/A | N/A |  |
|  | High vs low | 1.57 ( 1.21- 2.03) | N/A | N/A |  |
| Age at diagnosis | 55-64 vs <55 | 1.91 ( 1.35- 2.72) | 1.94 ( 1.23- 3.06) | 1.81 ( 0.96- 3.42) |  |
|  | 65-74 vs <55 | 3.99 ( 2.84- 5.60) | 4.40 ( 2.84- 6.82) | 3.66 ( 1.98- 6.77) |  |
|  | 75+ vs <55 | 7.82 ( 5.55-11.02) | 9.89 ( 6.36-15.40) | 6.57 ( 3.53-12.22) |  |
| Stage | 2 vs 1 | 0.82 ( 0.70- 0.96) | 0.97 ( 0.76- 1.24) | 0.77 ( 0.60- 0.98) |  |
| PSA at diagnosis | log-2 scale | 1.09 ( 1.01- 1.16) | 1.01 ( 0.92- 1.12) | 1.16 ( 1.03- 1.32) |  |
| PSA doubling time | <3 years | 0.96 ( 0.84- 1.08) | 0.97 ( 0.80- 1.18) | 1.13 ( 0.94- 1.36) |  |
|  | >=3 years | 0.85 ( 0.75- 0.96) | 0.76 ( 0.63- 0.92) | 1.01 ( 0.84- 1.22) |  |
|  | Unknown | 1.11 ( 0.95- 1.29) | 1.10 ( 0.85- 1.41) | 1.17 ( 0.94- 1.48) |  |
| Charlson score | 1 vs 0 | 1.40 ( 1.24- 1.58) | 1.32 ( 1.07- 1.62) | 1.53 ( 1.28- 1.82) |  |
|  | 2 vs 0 | 1.67 ( 1.42- 1.95) | 1.87 ( 1.49- 2.36) | 1.59 ( 1.24- 2.04) |  |
|  | 3+ vs 0 | 2.32 ( 1.96- 2.75) | 2.29 ( 1.77- 2.95) | 2.50 ( 1.92- 3.25) |  |
| Household median income | per $10,000 | 0.96 ( 0.94- 0.98) | 0.95 ( 0.92- 0.98) | 0.97 ( 0.94- 1.00) |  |

**Supplemental Table 4. Adjusted Cox model for prostate cancer-specific mortality by AUA risk categories (Fine-Gray competing risks model estimates).**

|  |  | **All (n=3925)** | **Low risk (n=2152)** | **Intermediate risk (n=1447)** | **High risk (n=326)** |
| --- | --- | --- | --- | --- | --- |
| **Variable** | **Level** | **Hazard Ratio (95% CI)** | | |  |
| Race/ethnicity | Asian first 10 yrs | 0.29 ( 0.09- 0.90) | 0.37 ( 0.05- 2.76) | 0.34 ( 0.08- 1.41) | No modeling was performed due to small number |
|  | Asian after 10 yrs | 5.41 ( 1.39-21.11) | 5.36 ( 0.53-54.36) | 3.56 ( 0.59-21.54) |  |
|  | Black | 1.06 ( 0.77- 1.46) | 1.50 ( 0.87- 2.60) | 0.90 ( 0.58- 1.42) |  |
|  | Hispanic | 0.73 ( 0.48- 1.10) | 0.79 ( 0.38- 1.63) | 0.68 ( 0.38- 1.23) |  |
| Gleason | 7 vs 6 | 1.76 ( 1.28- 2.43) | N/A | 2.12 ( 1.39- 3.23) |  |
| AUA risk strata | Intermediate vs low | 1.05 ( 0.72- 1.53) | N/A | N/A |  |
|  | High vs low | 1.15 ( 0.60- 2.22) | N/A | N/A |  |
| Age at diagnosis | 55-64 vs <55 | 1.27 ( 0.60- 2.71) | 1.48 ( 0.50- 4.43) | 0.89 ( 0.26- 3.06) |  |
|  | 65-74 vs <55 | 2.00 ( 0.96- 4.16) | 2.60 ( 0.87- 7.72) | 1.65 ( 0.52- 5.25) |  |
|  | 75+ vs <55 | 3.59 ( 1.72- 7.48) | 5.25 ( 1.77-15.52) | 2.66 ( 0.83- 8.50) |  |
| Stage | 2 vs 1 | 1.15 ( 0.77- 1.73) | 1.42 ( 0.66- 3.04) | 0.97 ( 0.53- 1.77) |  |
| PSA at diagnosis | log-2 scale | 1.26 ( 1.06- 1.51) | 1.07 ( 0.79- 1.45) | 1.56 ( 1.06- 2.29) |  |
| PSA doubling time | <3 years | 1.18 ( 0.86- 1.61) | 1.15 ( 0.61- 2.16) | 1.32 ( 0.85- 2.07) |  |
|  | >=3 years | 1.06 ( 0.77- 1.46) | 1.54 ( 0.89- 2.67) | 0.88 ( 0.54- 1.46) |  |
|  | Unknown | 1.33 ( 0.92- 1.90) | 1.91 ( 1.00- 3.64) | 1.40 ( 0.84- 2.35) |  |
| Charlson score | 1 vs 0 | 0.67 ( 0.47- 0.94) | 0.69 ( 0.37- 1.30) | 0.72 ( 0.45- 1.17) |  |
|  | 2 vs 0 | 0.64 ( 0.40- 1.01) | 0.47 ( 0.19- 1.18) | 0.89 ( 0.49- 1.62) |  |
|  | 3+ vs 0 | 0.79 ( 0.50- 1.24) | 0.78 ( 0.35- 1.74) | 0.81 ( 0.42- 1.56) |  |
| Household median income | per $10,000 | 0.97 ( 0.91- 1.03) | 0.97 ( 0.88- 1.07) | 0.97 ( 0.89- 1.06) |  |
